# sscNOVA: a semi-supervised convolutional neural network for predicting functional regulatory variants in autoimmune diseases

**DOI:** 10.3389/fimmu.2024.1323072

**Published:** 2024-02-06

**Authors:** Haibo Li, Zhenhua Yu, Fang Du, Lijuan Song, Yang Gao, Fangyuan Shi

**Affiliations:** ^1^ School of Information Engineering, Ningxia University, Yinchuan, China; ^2^ Collaborative Innovation Center for Ningxia Big Data and Artificial Intelligence Co-founded by Ningxia Municipality and Ministry of Education, Yinchuan, Ningxia University, Yinchuan, China; ^3^ School of Medical Technology, North Minzu University, Yinchuan, China

**Keywords:** autoimmune disease, regulatory variant, semi-supervised, deep learning, genome wide association studies

## Abstract

Genome-wide association studies (GWAS) have identified thousands of variants in the human genome with autoimmune diseases. However, identifying functional regulatory variants associated with autoimmune diseases remains challenging, largely because of insufficient experimental validation data. We adopt the concept of semi-supervised learning by combining labeled and unlabeled data to develop a deep learning-based algorithm framework, sscNOVA, to predict functional regulatory variants in autoimmune diseases and analyze the functional characteristics of these regulatory variants. Compared to traditional supervised learning methods, our approach leverages more variants’ data to explore the relationship between functional regulatory variants and autoimmune diseases. Based on the experimentally curated testing dataset and evaluation metrics, we find that sscNOVA outperforms other state-of-the-art methods. Furthermore, we illustrate that sscNOVA can help to improve the prioritization of functional regulatory variants from lead single-nucleotide polymorphisms and the proxy variants in autoimmune GWAS data.

## Introduction

Autoimmune disease (AD) is a type of disease in which the immune system mistakenly attacks the body’s own tissues and organs, resulting in symptoms such as myocarditis, skin rash, and joint pain, including asthma, type I diabetes, and systemic lupus erythematosus (1, 2). Family clustering of different autoimmune diseases suggests that genetic factors underlie common disease pathways (3), increasing the risk of certain autoimmune diseases by affecting the function of the immune system.

Recently, genome-wide association studies (GWAS) revealed that approximately 90% of disease-associated susceptibility variants are in noncoding regions (4). Now, we know that noncoding regions in the human genome harbor distinct regulatory elements, regulatory variants within these elements can potentially impact the regulation of gene expression (5), and hundreds of risk loci associated with autoimmune diseases have been identified (6)—for example, the G allele of the noncoding variant rs7216389 is associated with an increased risk of asthma (7). Although associations between variants and diseases can be identified (8), few regulatory variants were validated; it is still difficult to identify causal variants in autoimmune diseases (9).

Deep learning can now extract valuable information from complex genomic data, enabling the comprehension of regulatory variants linked to autoimmune diseases (10). Yousefian-Jazi et al. used a random forest model to identify regulatory variants associated with autoimmune diseases and studied their functionality, including the classification of putative causal variants for atopic dermatitis and inflammatory bowel disease (11). An integrated network-based approach called ARVIN was used to identify functional regulatory variants, and it was applied to seven autoimmune diseases (12). Lee et al. formulated the deltaSVM tool to predict several single-nucleotide polymorphisms (SNPs) associated with autoimmune diseases (13). Zhou et al. developed the ExPecto framework based on deep learning, enabling the prediction of mutation tissue-specific transcriptional effects, and experimentally validated predictions for four immune-related diseases (14). However, the data for functional regulatory variants in autoimmune diseases used by the previously mentioned tools is limited in quantity, either encompassing a smaller dataset or exclusively comprising variants from HGMD (15) and ClinVar (16). It is still difficult to systematically identify the function of regulatory variants in autoimmune diseases.

Given the lack of a “gold standard” dataset for functional regulatory variants, several unsupervised models were developed to identify functional regulatory variants, for example, MACIE (17), Eigen (18), and semi-supervised model GenoNet (19). Although unsupervised methods do not rely on labeled dataset, their capability may lag behind supervised methods when trained on a high-quality labeled dataset (17).

Here we develop sscNOVA, a semi-supervised convolutional neural network algorithm to identify functional regulatory variants from GWAS and eQTL dataset and explore the functional characteristics of regulatory variants in autoimmune diseases. We evaluate sscNOVA on the independent testing dataset and curated an experimentally validated testing dataset, and the results show that sscNOVA performs better than the state-of-the-art methods. sscNOVA could also identify the functional regulatory variants which are validated by the wet experiment and the candidate causal variants.

## Results

### Overview of sscNOVA

**Figure 1:**
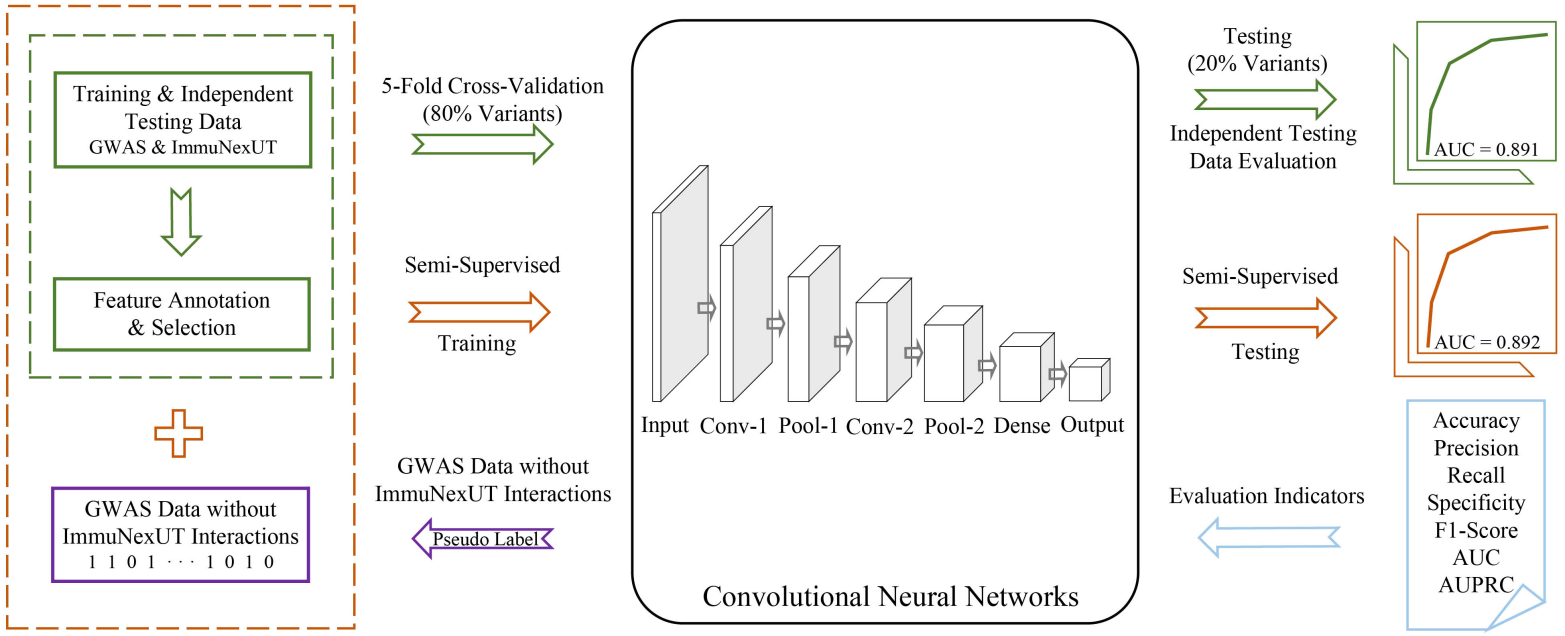
Overview of sscNOVA. sscNOVA takes VCF files as input and generates predicted probabilities for each variant as output. Among them, 80% of the intersection variants are designated as the training dataset (green solid box and arrow) for input into the convolutional neural network model (black solid box). The pre-training process employing a fivefold cross-validation training strategy, with 20% of the variants serving as an independent testing dataset for evaluating model performance (area under curve, AUC = 0.891, green curve). Based on the model’s predicted probability values, an optimal threshold is identified, and pseudo-labels are assigned to these unlabeled genome-wide association studies data without ImmuNexUT intersection variants (purple solid box and arrow). Subsequently, the dataset with pseudo-labels is merged with the original training dataset (yellow dashed box), and the model undergoes another round of fivefold cross-validation training. In this cross-validation process, the model with the highest AUC is referred to as sscNOVA. Notably, sscNOVA achieves an AUC of 0.892 on the independent testing dataset (yellow curve). The performance of sscNOVA is evaluated using seven metrics (blue section).

sscNOVA mainly includes the following modules: (1) acquiring and processing GWAS and ImmuNexUT data to construct the training data of sscNOVA, (2) 141 features related to 31 autoimmune diseases and 28 immune cell types are annotated by feature selection process, (3) training a supervised convolutional neural network (CNN) framework using GWAS and ImmuNexUT data and constructing a semi-supervised convolutional neural network framework (sscNOVA) with the GWAS data which do not have interactions with ImmuNexUT, and (4) evaluating the capability of the sscNOVA framework using GWAS and ImmuNexUT testing datasets as well as experimentally validated HGMD and ClinVar testing datasets (**Figure 1**).

### Feature annotation, selection, and analysis

**Figure 2:**
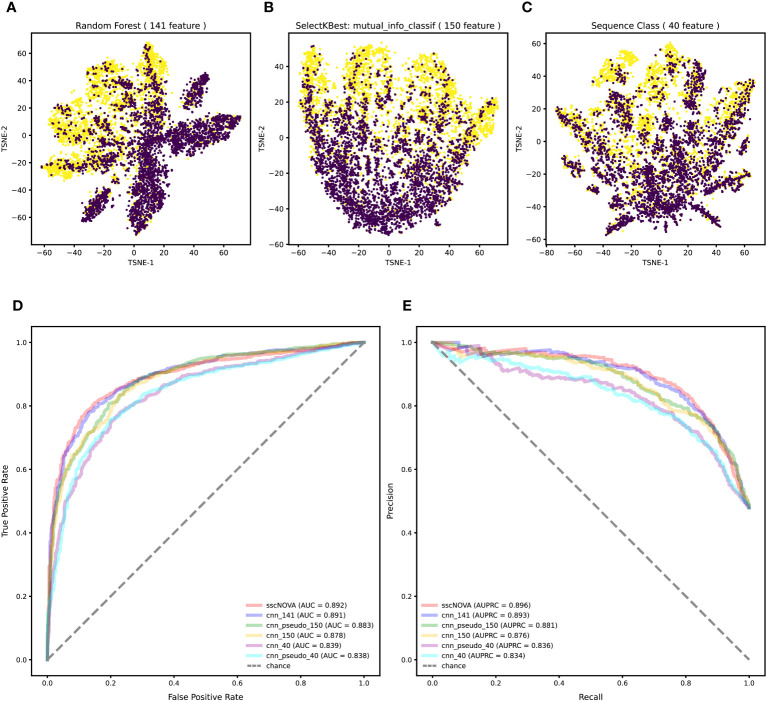
Feature selection and performance evaluation. **(A)** T-SNE plot of 141 features are chosen by the calculation of feature importance based on random forest. **(B)** T-SNE plot of 150 features selected by SelectKBest with mutual_info_classif method. **(C)** T-SNE plot of 40 features related to sequence classes which are provided by the Sei framework. **(D)** Comparison of the AUC between the 141, 150, and 40 features on the independent testing dataset with the convolutional neural network (CNN) model. **(E)** Comparison of the area under the precision–recall curve (AUPRC) between the 141, 150, and 40 features on the independent testing dataset with the CNN model.

Variants in the GWAS catalog that have a significant association with autoimmune diseases are unevenly distributed across different autoimmune diseases, especially variants associated with asthma and systemic lupus erythematosus (**Supplementary Figure 1**). Merging variants from the GWAS catalog and eQTLs with autoimmune diseases, we find that most of the positive variants are more likely to enrich in T helper cells, monocytes, and dendritic cells across 28 immune cell types (**Supplementary Figure 2**), which is consistent with what has been reported (20). To annotate all variants, we adopt 21,907 features by the Sei framework (21). Feature selection methods are employed to reduce the feature number, while the annotation features are redundant. Ultimately, 141 features were selected with top feature importance which was calculated based on random forest, 150 features were selected by SelectKBest with mutual_info_classif method, and 40 sequence class features were provided by the Sei framework (details in “Methods” section). The T-SNE plot shows that the classification effect of 141 features is better than that of 150 features and 40 features (**Figures 2A–C**).

To compare the three feature selection methods, we train the CNN with a training dataset to test the model performance on the independent testing dataset (details in “Methods” section). According to the model performance on the independent testing dataset, when using the 141 features, the CNN model performs the best, achieving an area under curve (AUC) of 0.891 and an area under the precision–recall curve (AUPRC) of 0.893, which demonstrates that using 141 features is superior to using 150 features and 40 features (**Figures 2D, E**). These results indicate that the proposed method based on the CNN model has better performance for predicting regulatory variants in autoimmune diseases when using 141 features (**Supplementary Figure 3**).

### Training and evaluation of sscNOVA

As the positive dataset in the CNN model only covers 10 autoimmune diseases, we adopt a semi-supervised learning approach to further improve the generalization ability of the model with the GWAS data which do not have interactions with the ImmuNexUT dataset (details in “Methods” section). As expected, sscNOVA shows an improvement in predictive performance on the independent testing dataset; its AUC and AUPRC are 0.892 and 0.896, respectively (**Figures 2D, E**).

**Figure 3:**
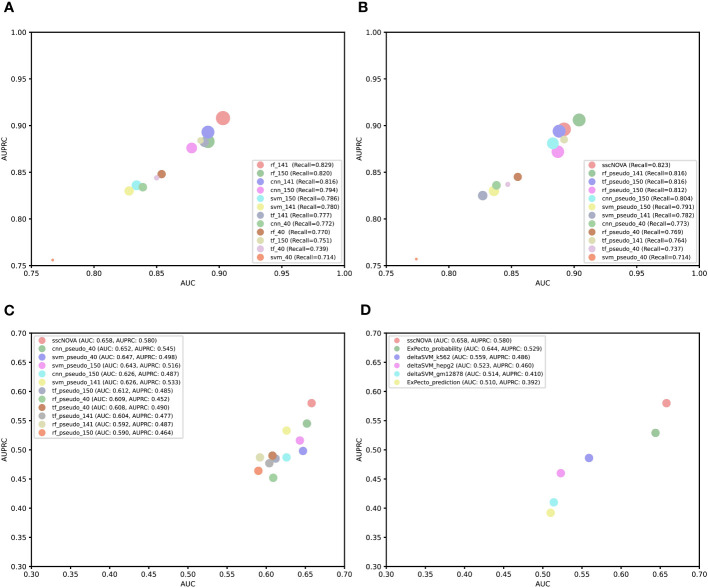
Comparison of performance among different models or tools. **(A)** Bubble plot of a different supervised model performance on the independent testing dataset. The x-axis is area under curve (AUC), the y-axis is area under the precision–recall curve (AUPRC), and the size of the bubble represents recall. **(B)** Bubble plot of a different semi-supervised model performance on the independent testing dataset. The x-axis is AUC, the y axis is AUPRC, and the size of the bubble represents recall. **(C)** Comparing convolutional neural network, support vector machine, random forest, and transformer algorithm models based on the experimentally curated testing dataset. The x-axis is AUC, and the y-axis is AUPRC. **(D)** Comparing sscNOVA, ExPecto, and deltaSVM tools based on the experimentally curated testing dataset. The calculation method involves weights for three types of cell lines for deltaSVM and employs two ExPecto score calculation methods. The x-axis is AUC, and the y-axis is AUPRC.

For the purpose of comparing the capability of CNN with other models, we construct three comparative models based on support vector machine (SVM), random forest, and transformer algorithms. Using the three types of features mentioned earlier, we apply the CNN model and these three models to perform fivefold cross-validation on the training dataset and evaluate their predictive performance on the independent testing dataset. According to the experimental results, we find that rf_141 achieves slightly higher AUC and AUPRC values, followed by the cnn_141 model (**Figure 3A**; **Supplementary Figure 4**). Afterward, we utilize the dataset containing pseudo-labeled data and train four models using identical methods. Though the AUC and AUPRC of sscNOVA on this dataset are slightly lower than rf_pseudo_141, sscNOVA still has the best recall (**Figure 3B**; **Supplementary Figure 5**). This suggests that sscNOVA is capable of accurately capturing features associated with positive variants, thereby reducing the risk of false negatives. This capability contributes to ensuring the effective identification of actual positive variants. The experimental results demonstrate that the pseudo-labeling method effectively alleviates the issue of limited labeled data and helps optimize the model’s predictive performance.

### Comparison on an experimentally curated testing dataset

To further validate the model performance, we use an experimentally curated testing dataset, in which positive variants include data from the HGMD and ClinVar databases (11), to evaluate four different models. Negative variants are obtained through three different methods: first, 190 negative variants are selected adjacent to positive variants (within ±1 kbp chromosomal positions); second, 118 negative variants are randomly selected from the human genome based on the chromosome numbers of positive variants; and third, 134 negative variants are selected adjacent to positive variants (within ±500 bp chromosomal positions). To compare the performance of the sscNOVA model on these three datasets, it is observed that the model performs best on the 190 negative variants selected adjacent to positive variants (**Supplementary Table 1**). Therefore, variants obtained through this method are chosen as the negative variants for the experimentally curated testing dataset. We observe that sscNOVA demonstrates excellent performance on both AUC and AUPRC metrics, ranking first (AUC = 0.658, AUPRC = 0.580) and showing significant improvement compared to the rf_141 model (**Figure 3C**; **Supplementary Figures 6, 7**). These results indicate that sscNOVA exhibits better generalization capabilities, allowing it to adapt better to new samples and data distributions. In addition, when training sscNOVA on the dataset containing pseudo-labeled data, the capability of sscNOVA on the experimentally curated testing dataset shows improvement in contrast to cnn_141 (**Supplementary Figures 6**, **7**). Moreover, we compare sscNOVA with existing tools for predicting regulatory variants in autoimmune diseases. We evaluate the capability of sscNOVA, ExPecto, and deltaSVM on the experimentally curated testing dataset (details in “Methods” section). Based on the experimental results, the sscNOVA model achieves better performance than the state-of-the-art methods in identifying regulatory variants in autoimmune diseases (**Figure 3D**).

### Prioritizing functional regulatory variants

**Figure 4:**
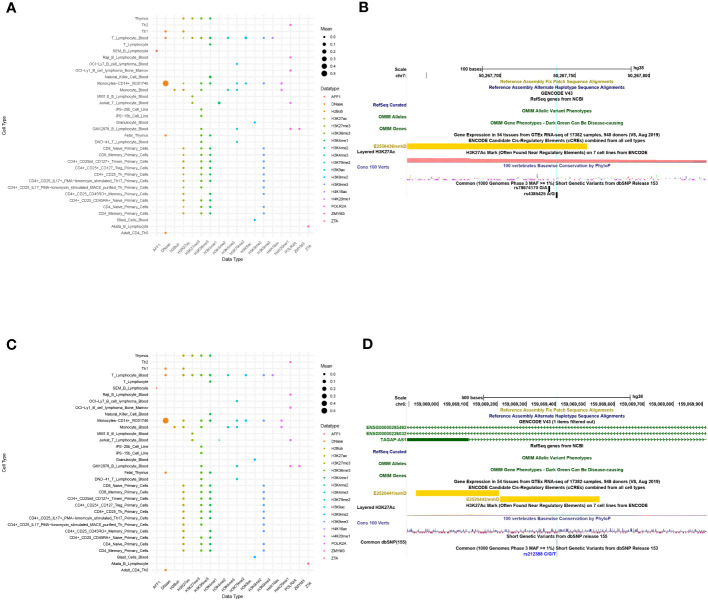
A total of 141 features of two variants, rs4385425 and rs212388, are produced in sscNOVA. **(A, C)** The 141 annotation features of variants rs4385425 and rs212388 with the same data type are merged with the average in each cell type to make the bubble plot. The x-axis is data type of annotations, and the y-axis is the cell type of annotations. **(B, D)** UCSC Genome Browser on Human with GRCh38 version is adopted to visualize the variants’ genome features.

The functional predictions of sscNOVA can be used to prioritize variants in GWAS. To illustrate the function of sscNOVA in this setting, we show two cases of variants with systemic lupus erythematosus and Crohn’s disease risk. The 213-bp open chromatin regions containing the variant rs4385425 targeted by CRISPR-CAS9 showed increasing IKZF1 (Ikaros) expression in Jurkat cells (22). This variant is proxy to the sentinel rs11185603 (*r* ^2^ = 0.99) associated with systemic lupus erythematosus. sscNOVA predicts this variant as positive, with a score 0.944. As shown in the UCSC Genome Browser (23), rs4385425 falls into the intergenic region and peak region of H3K27ac (**Figures 4A, B**). Compared with allele A, allele C improves the binding affinity of two active enhancer makers, H3K27ac and H3K4me1 (24), in multiple lymphocyte cells.

An additional functional regulatory variant is rs212388, which was found to be associated with Crohn’s disease. The authors show that the C allele of rs212388 has significantly lower levels of TAGAP mRNA in PBMCs. Moreover, data suggest that TAGAP deficiency was associated with infiltration and proinflammatory gene expression in CD4^+^ T cells (25). As shown in the UCSC Genome Browser, rs212388 falls into the intro region of TAGAP (**Figures 4C, D**). The features of rs212388 show that this variant has significant changes in the open chromatin features of CD4^+^ monocytes. The H3K27ac features in CD4^+^ lymphocytes also show differences between alleles of rs212388.

Overall, we investigate that sscNOVA could be used to predict the functional regulatory variants in autoimmune GWAS but also prioritize the proxy variants that link with lead SNPs.

## Methods

### Data acquisition and process

Autoimmune disease-related data are downloaded from the GWAS catalog with GRCh38 human reference genome. A total of 10,304 variants data are obtained, involving 31 autoimmune diseases such as asthma, rheumatoid arthritis, allergy, etc. The Immune Cell Gene Expression Atlas from the University of Tokyo (ImmuNexUT) data are downloaded from Ota M et al. (26) in the National Bioscience Database Centre (NBDC) website. This dataset includes two accession numbers, E-GEAD-398 and E-GEAD-420, which consist of expression quantitative trait loci (eQTLs) analysis data from 337 patients diagnosed with 10 different autoimmune diseases and 79 healthy volunteers, encompassing a total of 28 distinct immune cell subtypes. These datasets are used to identify associations between genetic variants and gene expressions. Among the datasets, E-GEAD-398 and E-GEAD-420 provide information on the correlation between gene expression levels and genotypes with 2,389,672 genetic variants records. E-GEAD-398 comprises variants with significant associations to autoimmune diseases, while E-GEAD-420 includes variants with non-significant associations to autoimmune diseases in addition to those found in E-GEAD-398. Take the intersection of variants associated with autoimmune diseases in E-GEAD-398 and GWAS as the positive variants of training dataset and independent testing dataset; for the corresponding negative variants, use the variants from E-GEAD-420.

#### Training dataset and independent testing dataset

The positive dataset was determined by taking the intersection of the processed GWAS catalog and ImmuNexUT numbered E-GEAD-398 variants to create 3,362 positive variants (**Supplementary Figure 8**). The negative dataset is created by selecting variants with a *P*-value greater than 0.1 and an allele frequency (AF) greater than 0.3 in ImmuNexUT data numbered E-GEAD-420, resulting in 3,670 negative variants (**Supplementary Figure 8**). After merging the positive dataset with the negative dataset, we randomly sampled the variants’ data and split it into training and independent testing dataset in an 80% is to 20% ratio, as the 20% independent testing dataset does not participate in any model training process.

#### Experimentally curated testing dataset

We use the 140 positive variants utilized by Yousefian-Jazi et al. (https://github.com/jieunjung511/Autoimmune-research) (11). These variants come from HGMD and ClinVar, and a total of 118 positive variants conforming to the VCF format are obtained. Subsequently, we screen the variants within 1 kbp upstream and downstream of the chromosomal positions where the 118 positive variants are located, calculate the conservation values of these variants, and only retain the variants with a phastcons100way conservation value less than 0.5 and AF greater than 0.3. Therefore, the final experimentally curated testing dataset contains 118 positive variants and 190 negative variants (**Supplementary Figure 8**). In addition, we employ additional methods to obtain negative variants. One approach involves using a pseudo-random number generator on the GRCh37 genome to randomly select chromosomes and positions. This ensures that the chosen positions are not adjacent to known positive variants, resulting in the generation of 118 negative variants. The other method involves choosing 134 negative variants located within ±500 bp chromosomal positions adjacent to the positive variants.

### Feature annotation and selection

After annotating the variants with 21,907 features from the Sei framework, feature selection is carried out to select the most informative and relevant features for the analysis, thus focusing on those that are more likely to be associated with the phenotype of interest or have potential functional significance (27).

Initially, 3,102 features related to immune cells are selected from the 21,907 features. Next, two methods, mutual_info_classif and f_classif of SelectKBest, are used to select 1,000, 800, 600, 400, and 200 features from the 3,102 immune-related cell features, respectively (**Supplementary Figure 9**). Mutual_info_classif method of SelectKBest shows better classification performance than f_classif (**Supplementary Figure 10**). Subsequently, we continue using mutual_info_classif to select 150, 100, and 50 features from the 3,102 immune-related cell features.

Additionally, we use the feature importance which was calculated based on random forest to select 141 features (**Supplementary Figure 10**). Three groups of features are compared by the performance trained with random forest model, which includes the 150 features selected by SelectKBest, 141 features selected by the top feature importance which was calculated based on random forest, and 40 features of sequence classes provided by the Sei framework. The T-distributed stochastic neighbor embedding (t-SNE) (28) plot shows that the classification performance is better with 141 features selected by using the random forest method (**Supplementary Figure 10**). Upon validation using the random forest model, the AUC and AUPRC based on the 141 features selected outperform those selected by other methods (**Supplementary Figure 11**). The mutual_info_classif method is superior to the f_classif method (details in **Supplementary Table 2**).

### Method for constructing a pseudo-labeled dataset

We construct a pseudo-labeled dataset based on autoimmune disease-related GWAS data which do not have interactions with ImmuNexUT using a threshold and *t*-test method. First, we use the cnn_141 model to predict the probability of the GWAS data without ImmuNexUT interactions and subject them to a fivefold cross-validation. For each variant, five probability values are generated as predictions. First, the Student’s *t*-test (29) is conducted to determine if the differences between these five probability values for each variant are statistically significant, with a *P*-value less than 0.05. If the *P*-value of this variant is less than 0.05, the variant is retained; otherwise, it is discarded. To find the optimal pseudo-label threshold for this variant, a parameter search is conducted. Then, using a threshold of 0.5 as a reference, we create five groups of thresholds with ±0, ± 0.1, ± 0.2, ± 0.3, and ±0.4 for all unlabeled variants. (**Supplementary Figure 12**). Next, we utilize the variants with pseudo-labeled data and the original training dataset to retrain the model and compare the models’ performance. Through this approach, we identify the optimal threshold for applying pseudo-labels, which involves considering cnn_141 model-predicted probabilities greater than 0.9 as positive variants and those less than 0.1 as negative variants. In the end, we filter out 2,759 positive variants and 626 negative variants from 6,924 variants data, discarding 3,539 variants that did not satisfy the criteria.

### Method for constructing a semi-supervised model

The approach to constructing sscNOVA involves using a trained model to predict variants from the GWAS data which do not have interactions with ImmuNexUT and then pseudo-labeling the unlabeled GWAS data using a threshold and *t*-test method. After that, we merge the dataset with pseudo-labeled data and the original training dataset and evaluate the model’s capability using AUC on the independent testing dataset. The threshold corresponding to the highest AUC is selected as the final pseudo-labeling method. Using the same methods, we retrain the models with the augmented dataset.

### Semi-supervised model architecture

Semi-supervised learning is a learning approach that combines supervised and unsupervised learning (30). In the presence of a small amount of labeled data, semi-supervised models infer the structure and features of unlabeled data to perform classification and prediction tasks, thereby enhancing model performance with limited labeled data (31). The semi-supervised sscNOVA model implementation consists of the following eight layers:

1. First convolutional layer: Let *x* be the input feature of length 141 and *W* be the convolutional kernel of size 5. The output *y* of the convolutional layer can be calculated as Equation 1:


(1) $$\begin{array}{c} y_{i}=GELU(\Sigma_{j=0}^{4}W_{j}\cdot x_{i+j}+b) \end{array}$$

where *i* ranges from 0 to 136, and *b* is the bias term. The resulting output *y* will have a shape of (137, 32), the number 32 of which represents the quantity of distinct kernels applied to the input data.

2. First max-pooling layer: Given the (137, 32) output shape from the prior Conv1D layer, applying a max-pooling operation with a pool size of 2 reduces each feature map’s length by half while keeping 32 feature maps. The output *z* of the max-pooling layer can be calculated by taking the maximum value within every consecutive two elements in each feature map as Equation 2:


(2) $$\begin{array}{c} z_{i,j}=max(y_{2i,j},y_{2i+1,j}) \end{array}$$

where *i* ranges from 0 to 67, and *j* ranges from 0 to 31. The resulting output *z* will have a shape of (68, 32).

3. Second convolutional layer: Let *y* be the previous output of shape (68, 32) and *W'* be the convolutional kernel of size 5 for the second convolutional layer, where the number of kernels is 64. The output *z* can be calculated as Equation 3:


(3) $$\begin{array}{c} z_{i,j}=GELU(\Sigma_{k=0}^{4}W_{k}^{'}\cdot y_{i+k,j}+b^{\prime }) \end{array}$$

where *i* ranges from 0 to 63, *j* ranges from 0 to 63, *k* ranges from 0 to 4, and *b'* is the bias term. The resulting output *z* will have a shape of (64, 64).

4. Second max-pooling layer: The output *w* of the second max-pooling layer can be calculated similarly to the first pooling layer as Equation 4:


(4) $$\begin{array}{c} W_{i,j}=max(z_{2i,j},z_{2i+1,j}) \end{array}$$

where *i* ranges from 0 to 31, and *j* ranges from 0 to 63. The resulting output *w* will have a shape of (32, 64).

5. Flattening layer: The flattening operation reshapes the 2D array *w* into a 1D array *v* by concatenating its rows as Equation 5:


(5) $$\begin{array}{c} v_{k}=w_{i,j} \end{array}$$

where *k* = *i*×64+*j*, and *k* ranges from 0 to 2,047. The resulting output *v* will have a shape of (1, 2,048).

6. Fully connected (dense) layer: Let *v* be the input vector of size 2,048 and *W"* be the weights of the dense layer. The output *x* of the dense layer can be calculated as Equation 6:


(6) $$\begin{array}{c} x_{i}=GELU(\Sigma_{j=0}^{2047}W_{j,\text{i}}^{''}\cdot v_{j}+b_{i}^{''}) \end{array}$$

where *i* ranges from 0 to 15 and corresponds to the 16 specified units in the dense layer, *j* ranges from 0 to 2,047, and $b_{i}^{''}$ is the bias term. The resulting output *x* will have a shape of (16), which matches the number of units within the layer.

7. Dropout layer: The dropout layer performs an element-wise multiplication by a binary mask to apply dropout as Equation 7:


(7) $$\begin{array}{c} y_{i}=x_{i}\cdot m_{i} \end{array}$$

where *i* ranges from 0 to 15, and *m_i_* is a binary mask randomly set to 0 or 1 with a probability of 0.1.

8. Output dense layer: Let *y* be the output of the dropout layer and *W'''* be the weights of the output dense layer. The final output *z* can be calculated as Equation 8:


(8) $$\begin{array}{c} z=\sigma (\Sigma_{\text{i}=0}^{15}W_{i}^{'''}\cdot y_{i}+b^{\prime \prime \prime }) \end{array}$$

where *σ* is the sigmoid activation function, and *b'''* is the bias term.

The model’s architecture is configured for training by utilizing the “binary_crossentropy” loss function (BCELoss). The loss function is as follows Equation 9:


(9) $$\begin{array}{c} BCELoss=-\frac{1}{N}\Sigma_{\text{i}=1}^{N}(y_{i}log(p_{i})+(1-y_{i})log(1-p_{i})) \end{array}$$

where *N* is the number of variants, *y_i_* represents the actual label (0 or 1) of variant *i*, and *p_i_* represents the predicted probability by the model that variant *i* belongs to the positive class. In this loss function, the term *y_i_log*(*p_i_*) penalizes the model for inaccuracies when predicting positive variants, while (1–*y_i_*)*log*(1–*p_i_*) penalizes inaccuracies in predicting negative variants. The objective of the model is to minimize this loss function to make its predictions closer to the actual labels.

In this neural network model, we opt to use Gaussian Error Linear Unit (GELU) (32) as the activation function, and it is applied in both the convolutional layers and the fully connected layers. Additionally, the “Adam” optimizer is adopted as the guiding algorithm responsible for the model’s weight updates throughout the training process. Utilizing its default learning rate of 0.001, the Adam optimizer dynamically adjusts the learning rates for individual parameters (33). The training is conducted in 50 epochs.

### sscNOVA functional significance score

For each variant *i*, *y_prob_* [*i*] is a probability value between 0 and 1, representing the model’s prediction of the probability that it belongs to the positive class. Therefore, the scoring formula can be expressed as Equation 10:


(10) $$\begin{array}{c} f_{score}(i)=y_{prob}(i)=dense(flatten(pool(conv(i)))) \end{array}$$

where *i* represents the *i*–th variant in the dataset; *conv*, *pool*, *flatten*, and *dense* represent one-dimensional convolution operation, maximum pooling operation, pooling result flattening, and full connection operation, respectively; and *f_score_* (*i*) represents the predicted probability of the *i*–th variant belonging to the positive class. The aim is to determine a threshold that achieves a balanced trade-off between these rates within the context of the specific dataset’s characteristics, where values above the threshold are classified as positive and values below the threshold are classified as negative.

### sscNOVA comparison with ExPecto and deltaSVM

When comparing with ExPecto, we try two methods to calculate the scores. The first method involves comparing the predicted variants labels from the ExPecto model with the true labels and then computing the evaluation metrics based on this comparison. Among them, ExPecto employs a minimum predictive effect threshold (>0.3), which is a threshold for log fold-change recommended by the official website (https://hb.flatironinstitute.org/expecto/about). The second method involves taking the absolute values of the ExPecto model’s predicted probabilities and then normalizing and calculating the evaluation metrics based on the normalized probabilities and the true labels. To calculate the deltaSVM scores, the GM12878, K562, and HepG2 cell line models developed by deltaSVM are all tested.

## Discussion

Identifying the functional impact of regulatory variants related to autoimmune diseases is a significant challenge in human genetics (34). Due to the scarcity of experimentally validated functional regulatory variants in autoimmune diseases, we adopt the idea of semi-supervised learning, combining labeled and unlabeled data, to develop a framework based on convolutional neural network algorithms to predict functional regulatory variants in autoimmune diseases. sscNOVA provides a feasible solution for the problem of limited gold standard data for regulatory variants in autoimmune diseases. By utilizing the information from unlabeled data, our algorithm helps the models gain more comprehensive information and further elevates the predictive performance. Moreover, the current model results represent the optimal model obtained after fine-tuning (**Supplementary Table 3**, **4**).

Since sscNOVA is based on sequence prediction, it can predict various types of variants. To test whether sscNOVA can help find the rare variants or the variants have not been observed, we utilize the sscNOVA model to predict the validated rare or not previously observed variants in two studies in which the variants were validated by the MPRA assays (35, 36). The recall and AUC values in HeLa, LNCaP, and NPC cell lines indicate that sscNOVA has potential for identifying rare variants (**Supplementary Figure 13**). In contrast to traditional supervised learning methods, the idea of semi-supervised learning allows us to effectively utilize unlabeled samples in the presence of limited labeled samples, overcoming issues related to data sparsity and missing sample labels (37).

However, some challenges also exist—for instance, the insufficient number of experimentally validated functional regulatory variants may introduce label noise during model training (38), thus reducing prediction performance. It is expected that an increasing amount of experimentally validated variants data will become available, which can intensify prediction performance by leveraging high-confidence data. Due to the limited number of experimentally validated variants in autoimmune diseases, there is a decline in performance on the experimentally curated testing dataset. We localize the positional information of variants in both the independent testing dataset and the experimentally curated testing dataset. Additionally, we conduct a categorized analysis to assess the predictive capability of sscNOVA for each positional category. (**Supplementary Figures 14A, B** and **Supplementary Table 5**). We find that sscNOVA has better performance with variants falling into the intron and promoter regions, but variants in the intergenic regions might be missed out by sscNOVA. The annotations in intron and promoter regions are more abundant than those in intergenic regions, which may make it easier for the model to learn patterns of intron variants during the training phase (39, 40). Meanwhile, integrating more experimental validation and functional regulatory variants data will provide greater opportunities to improve predictive performance.

Furthermore, in the ever-evolving field of deep learning, there may be better feature annotation tools capable of capturing the interactions between regulatory regions more effectively. By combining appropriate feature selection methods and training strategies, it could improve the prediction of functional regulatory variants in autoimmune diseases and enhance the capability of model (41). In conclusion, a model based on semi-supervised deep learning can provide new insights and directions for the study of autoimmune diseases, facilitating further investigation into the pathogenesis of autoimmune diseases.

## Data availability statement

The autoimmune diseases related GWAS data can be downloaded from https://www.ebi.ac.uk/gwas/docs/file-downloads (Version: All associations v1.0). The ImmuNexUT data can be downloaded from https://humandbs.biosciencedbc.jp/en/hum0214-v6. The source code and detail documentation of sscNOVA are available at https://github.com/NXU-Shilab/sscNOVA.

## Author contributions

HL: Formal analysis, Methodology, Visualization, Writing – original draft. ZY: Formal analysis, Funding acquisition, Validation, Writing – review & editing. FD: Data curation, Funding acquisition, Writing – review & editing. LS: Data curation, Funding acquisition, Writing – review & editing. YG: Formal analysis, Writing – review & editing. FS: Conceptualization, Formal analysis, Funding acquisition, Supervision, Writing – original draft, Writing – review & editing.
