# Supplementary material for: sscNOVA: a semi-supervised convolutional neural network for predicting functional regulatory variants in autoimmune diseases
Source: Front Immunol. 2024 Feb 6;15:1323072. doi: 10.3389/fimmu.2024.1323072 (PMC10876991; doi:10.3389/fimmu.2024.1323072)
Supplement: Supplementary file 1 [file DataSheet_1.docx]

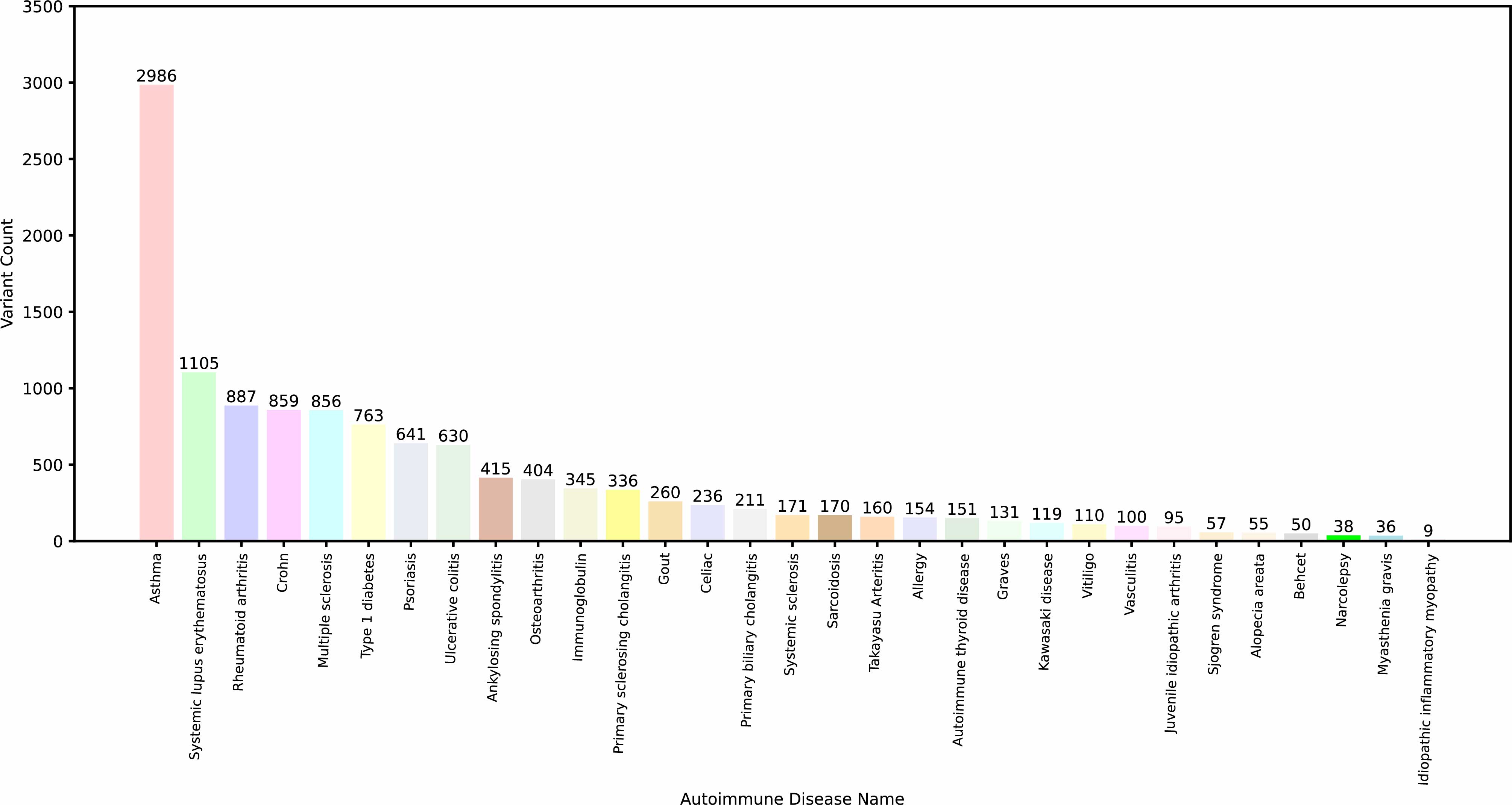


**Supplementary Figure 1.** Overview of variants number in each type of diseases in GWAS Catalog. Counting the number of variants in 31 immune-related diseases in GWAS Catalog. The x axis is the type of disease, the y axis is the count of variant.


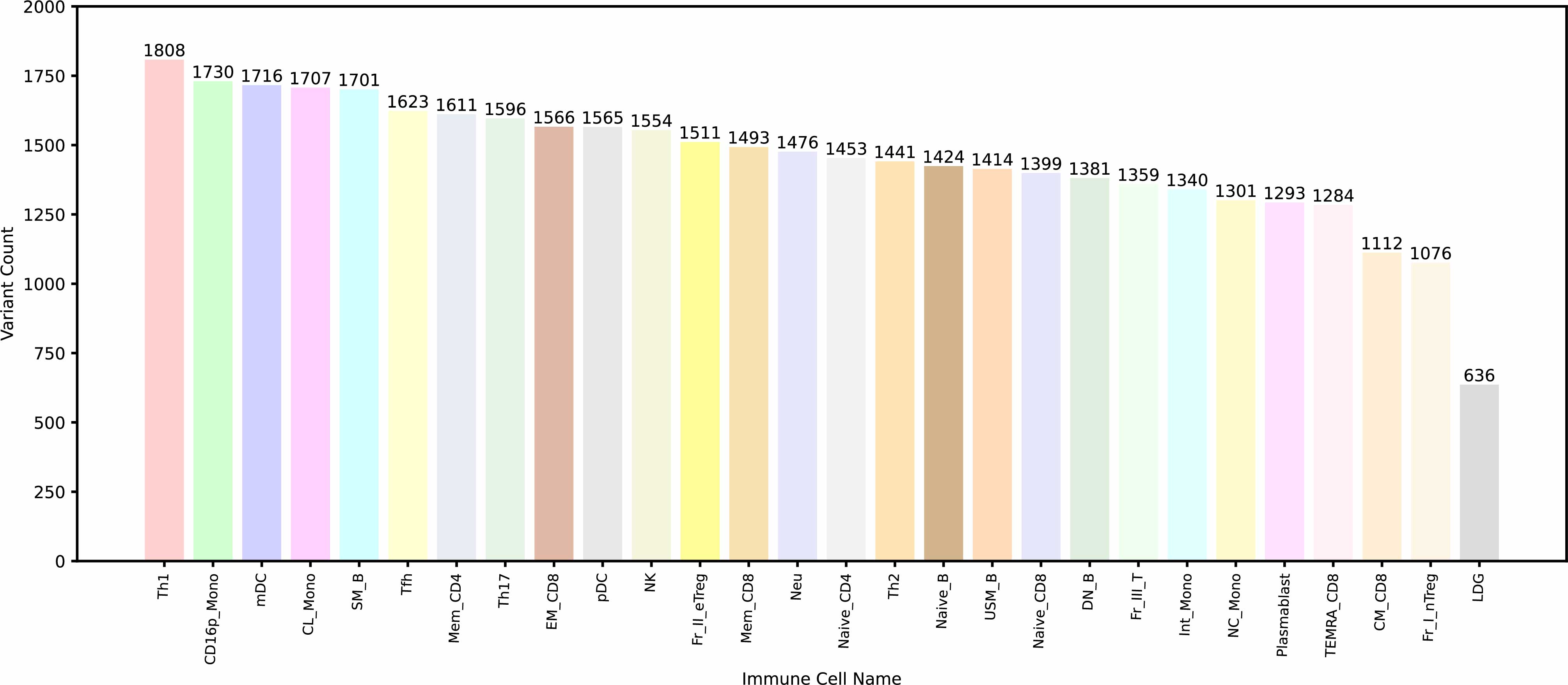


**Supplementary Figure 2.** Overview of variants distribution in each type of cells in positive dataset. Counting the distribution of variants in 28 immune-related cells in positive dataset. These 28 immune-related cells are from ImmuNexUT. The x axis is the type of cell, the y axis is the count of variant.


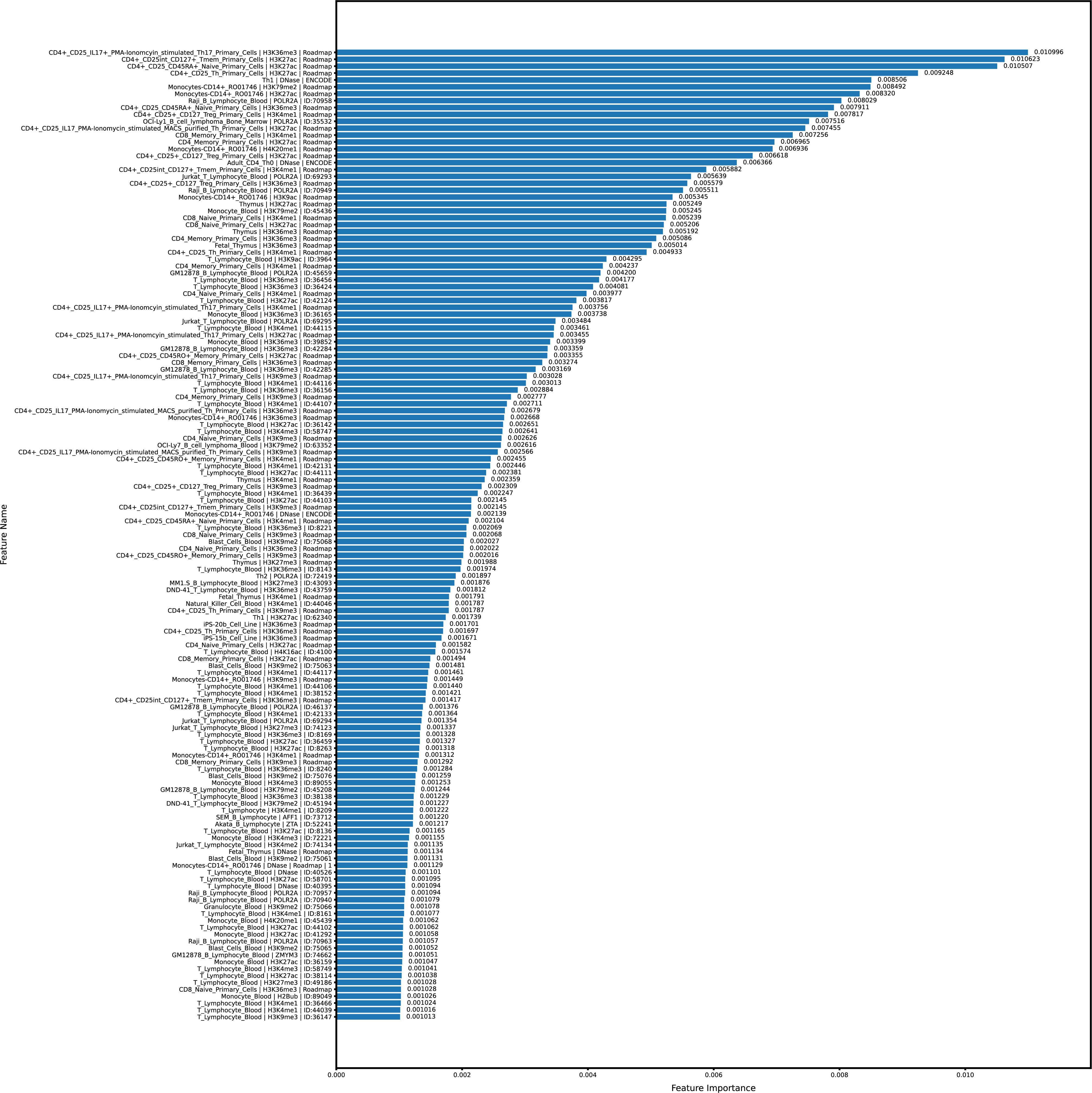


**Supplementary Figure 3.** Bar plot of feature importance score based on Random Forest. Including a sorted order 141 features along with its corresponding feature importance score. The x axis is the score of feature importance, the y axis is the name of feature.


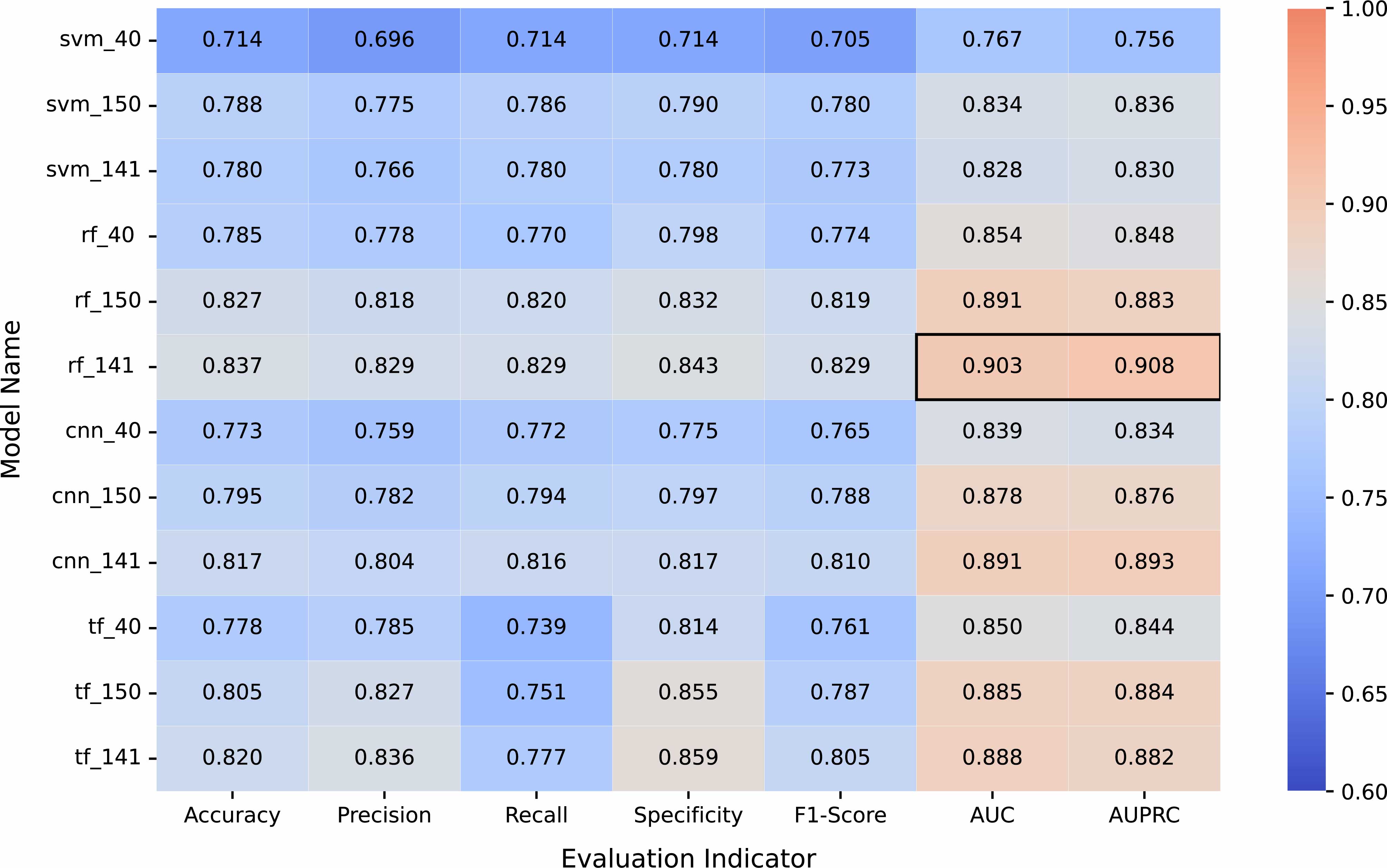


**Supplementary Figure 4.** Heatmap of results on the independent testing dataset by different supervised models. Including metrics such as Accuracy, Precision, Recall, Specificity, F1-Score, AUC, and AUPRC. The x axis is evaluation indicator, the y axis is model.


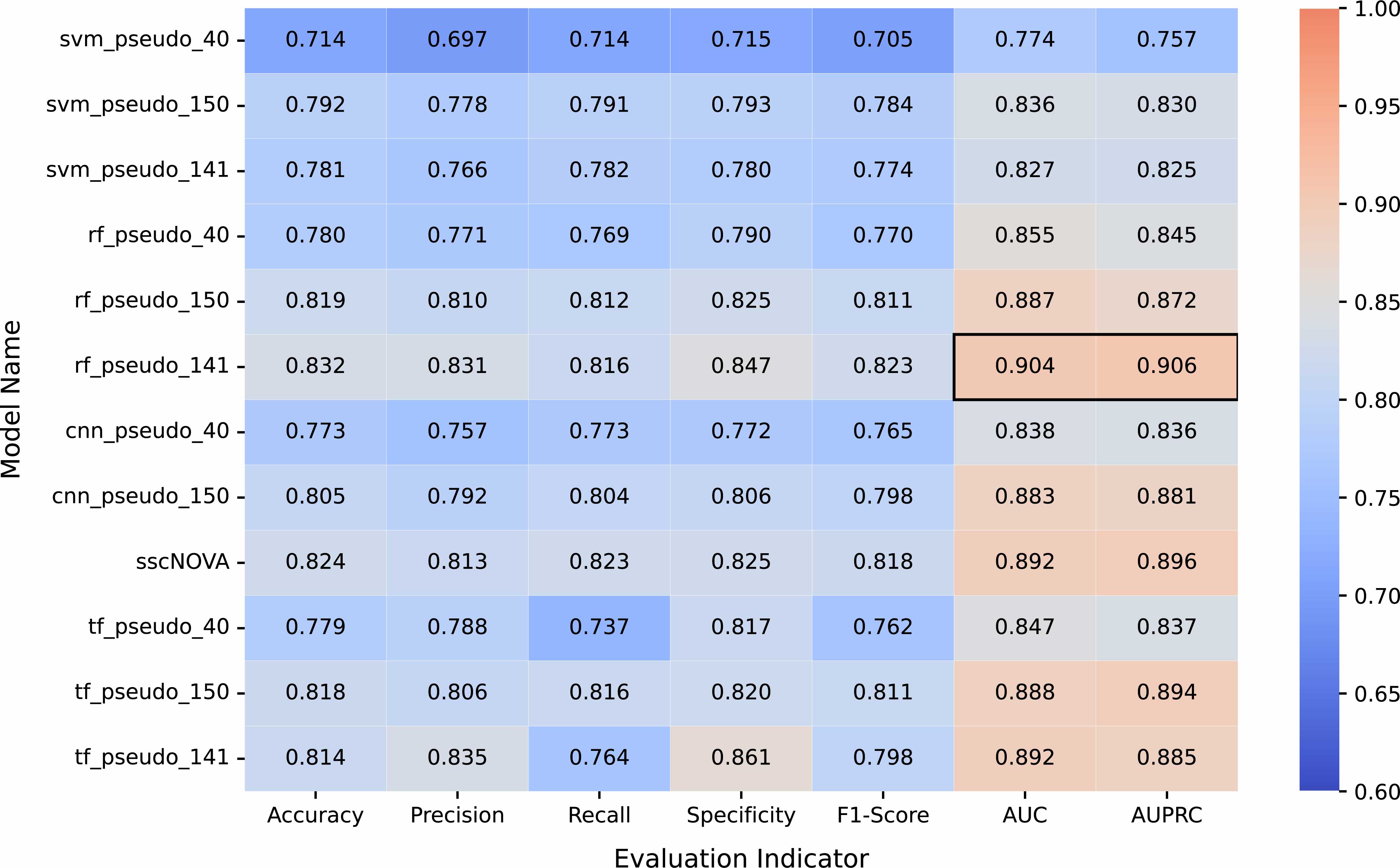


**Supplementary Figure 5.** Heatmap of results on the independent testing dataset by different semi-supervised models. Including metrics such as Accuracy, Precision, Recall, Specificity, F1-Score, AUC, and AUPRC. The x axis is evaluation indicator, the y axis is model.


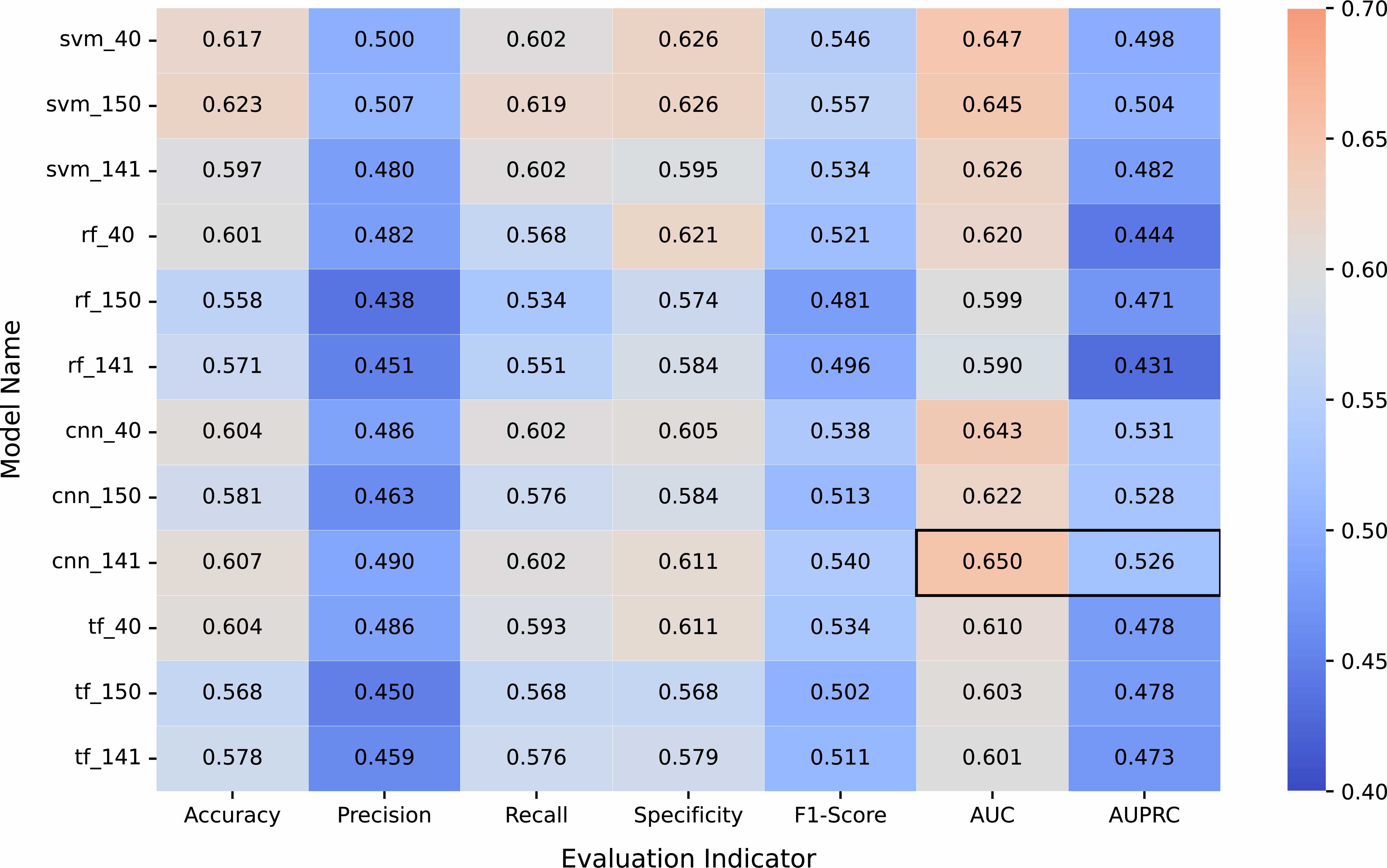


**Supplementary Figure 6.** Heatmap of results on the experimentally curated testing dataset by different supervised models. Including metrics such as Accuracy, Precision, Recall, Specificity, F1-Score, AUC, and AUPRC. The x axis is evaluation indicator, the y axis is model.


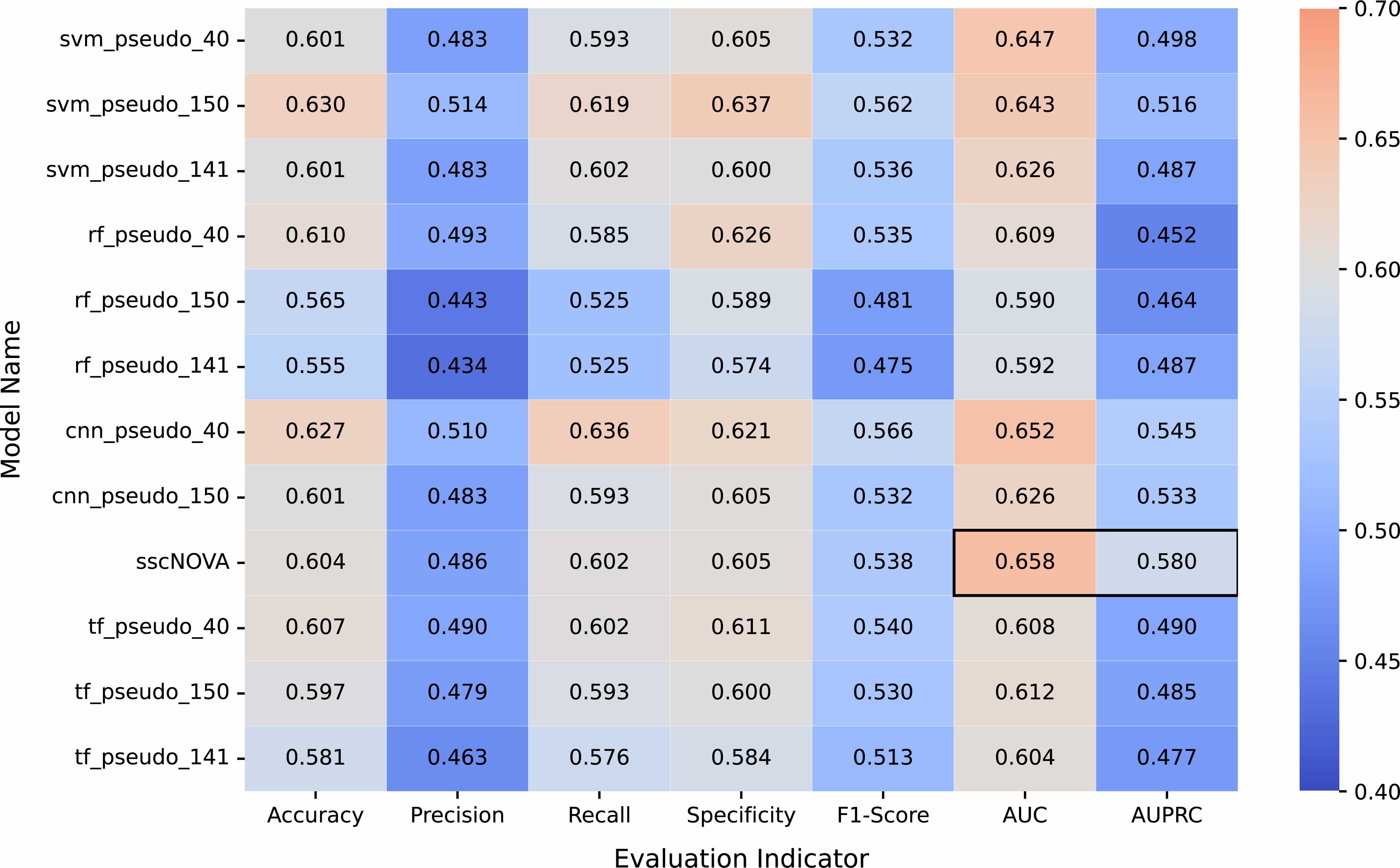


**Supplementary Figure 7.** Heatmap of results on the experimentally curated testing dataset by different semi-supervised models. Including metrics such as Accuracy, Precision, Recall, Specificity, F1-Score, AUC, and AUPRC. The x axis is evaluation indicator, the y axis is model.


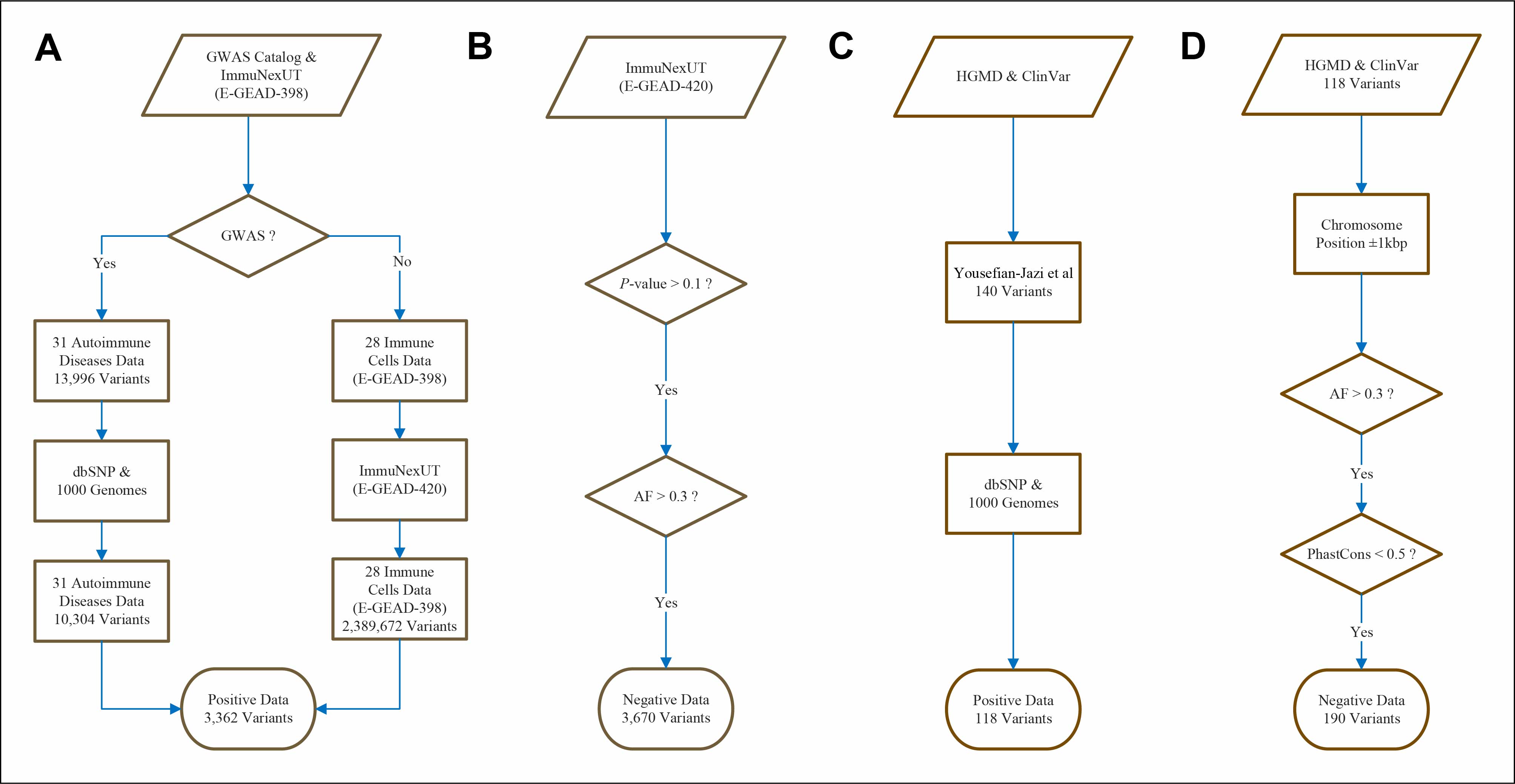


**Supplementary Figure 8.** Flowchart of data processing. (A) Processing GWAS Catalog variants based on dbSNP human_9606_b151 and 1000 Genomes phase 3 databases and handling the E-GEAD-398 variants to VCF format based on the E-GEAD-420 variants from ImmuNexUT. Taking the processed GWAS Catalog variants and the intersection variants between ImmuNexUT's E-GEAD-398 as the positive variants for training and independent testing dataset. (B) Selecting variants from ImmuNexUT's E-GEAD-420 with *P*-value > 0.1 and AF > 0.3 as negative variants for training and independent testing dataset. (C) Processing variants in curated experimentally validated dataset as positive variants. (D) Extracting variants from the chromosome regions located 1kbp upstream and downstream of the variants obtained in (C). Filter the selected variants to include those with AF > 0.3 and PhastCons < 0.5 as negative variants for experimentally curated testing dataset.


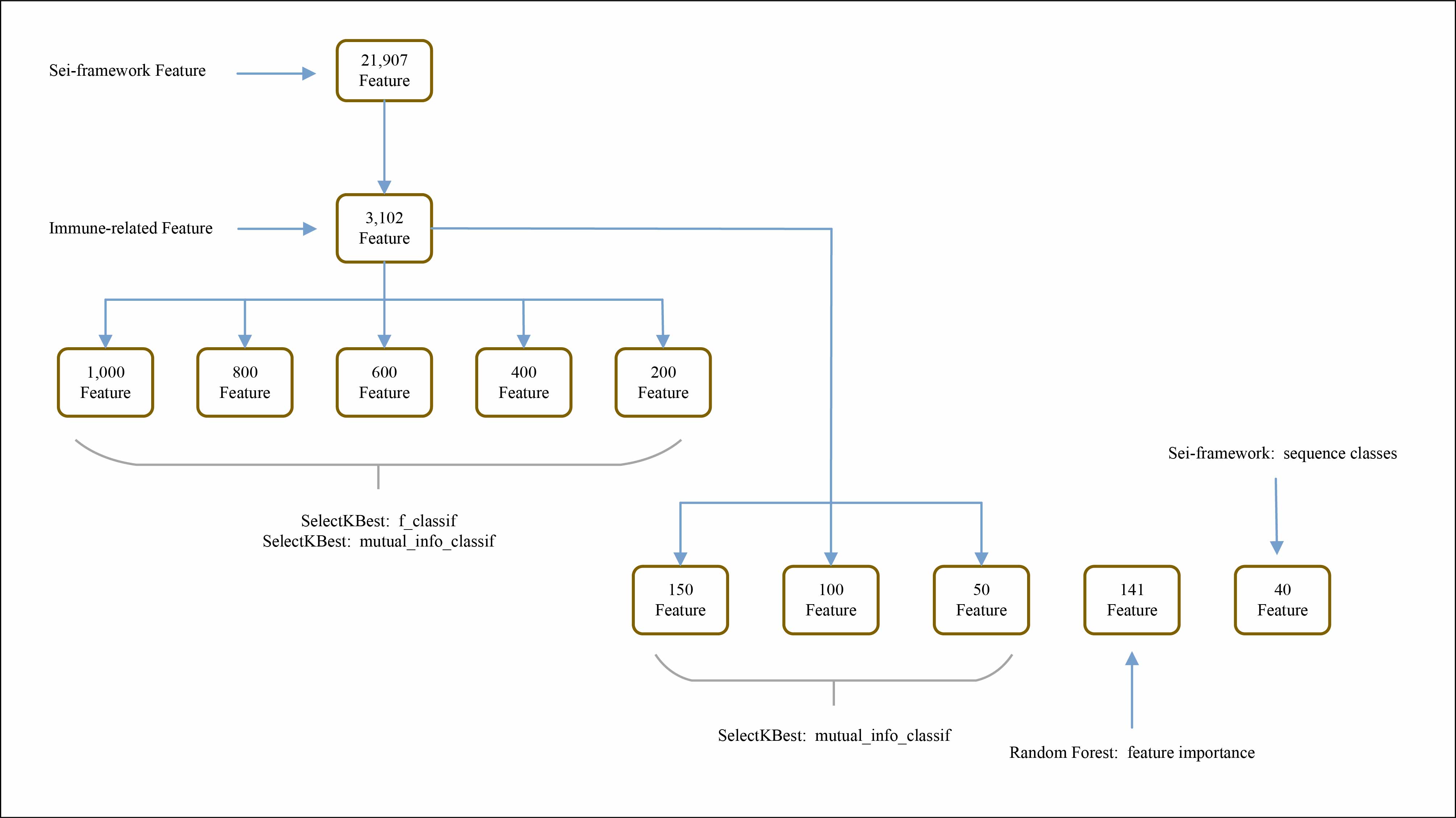


**Supplementary Figure 9.** Feature annotation and selection process. 3,102 immune-related cells features are selected from the 21,907 features, F_classif and mutual_info_classif of SelectKBest are used to select 1,000, 800, 600, 400, and 200 features from the 3,102 immune-related cells features, respectively. Mutual_info_classif of SelectKBest are utilized 150, 100, and 50 features from the 3,102 immune-related cells features. 141 features selected with feature importance which calculated based on Random Forest and 40 sequence classes features provided by the Sei framework.





**Supplementary Figure 10.** Feature visualization results by t-SNE. These include 21,907 features from the Sei framework, 3,102 features specifically related to immune cells, as well as 1,000, 800, 600, 400, and 200 features respectively selected using the mutual_info_classif and f_classif methods within SelectKBest. In addition, there are 150, 100, and 50 features selected by mutual_info_classif method within SelectKBest, as well as 141 features selected with feature importance based on Random Forest.


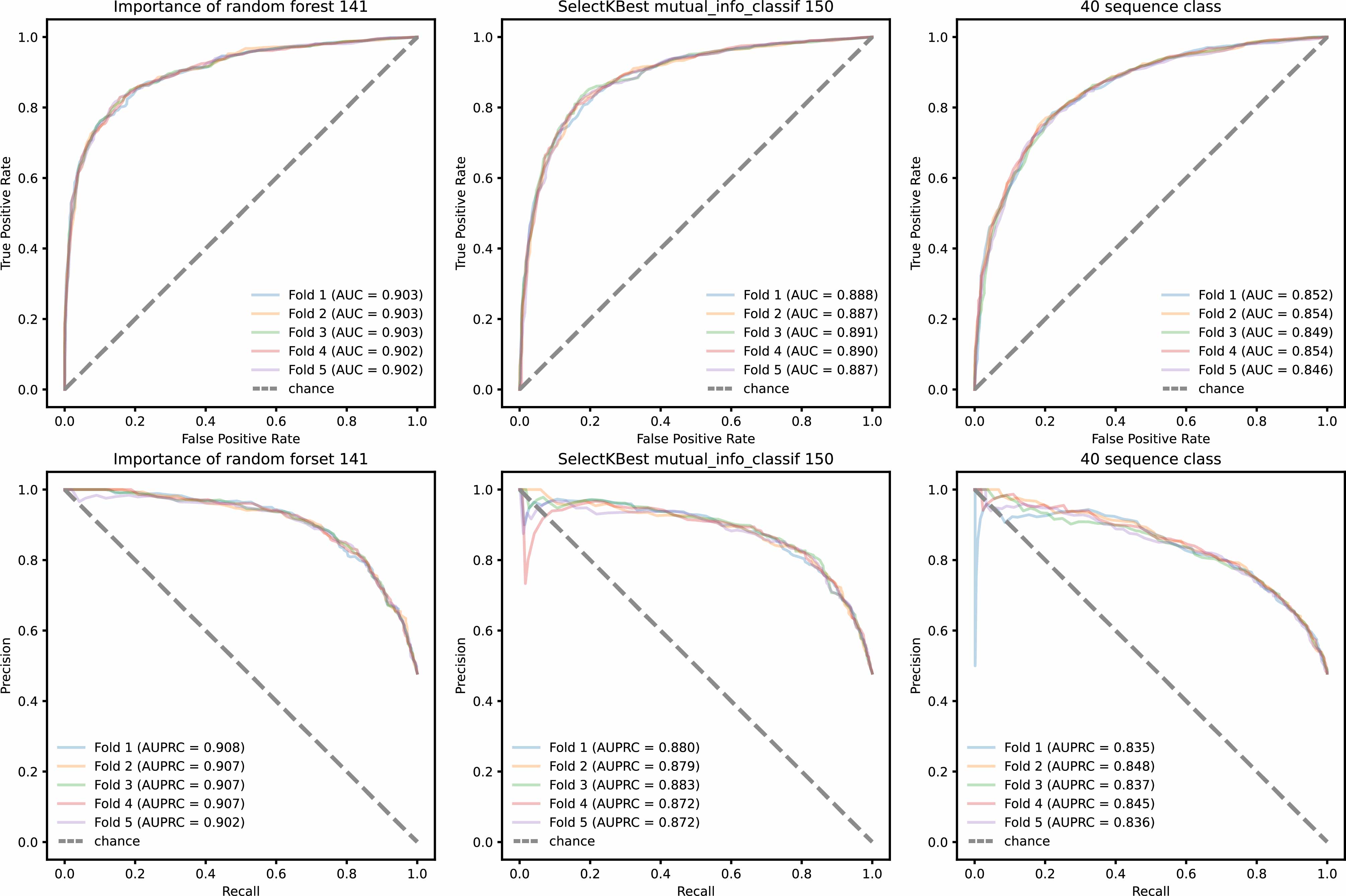


**Supplementary Figure 11.** Comparison of the AUC and AUPRC between the 141, 150 and 40 features. The random forest model is subjected to five-fold cross-validation for AUC and AUPRC using 141, 150 and 40 features. Among these features, the random forest model trained with 141 features performs better than the others.


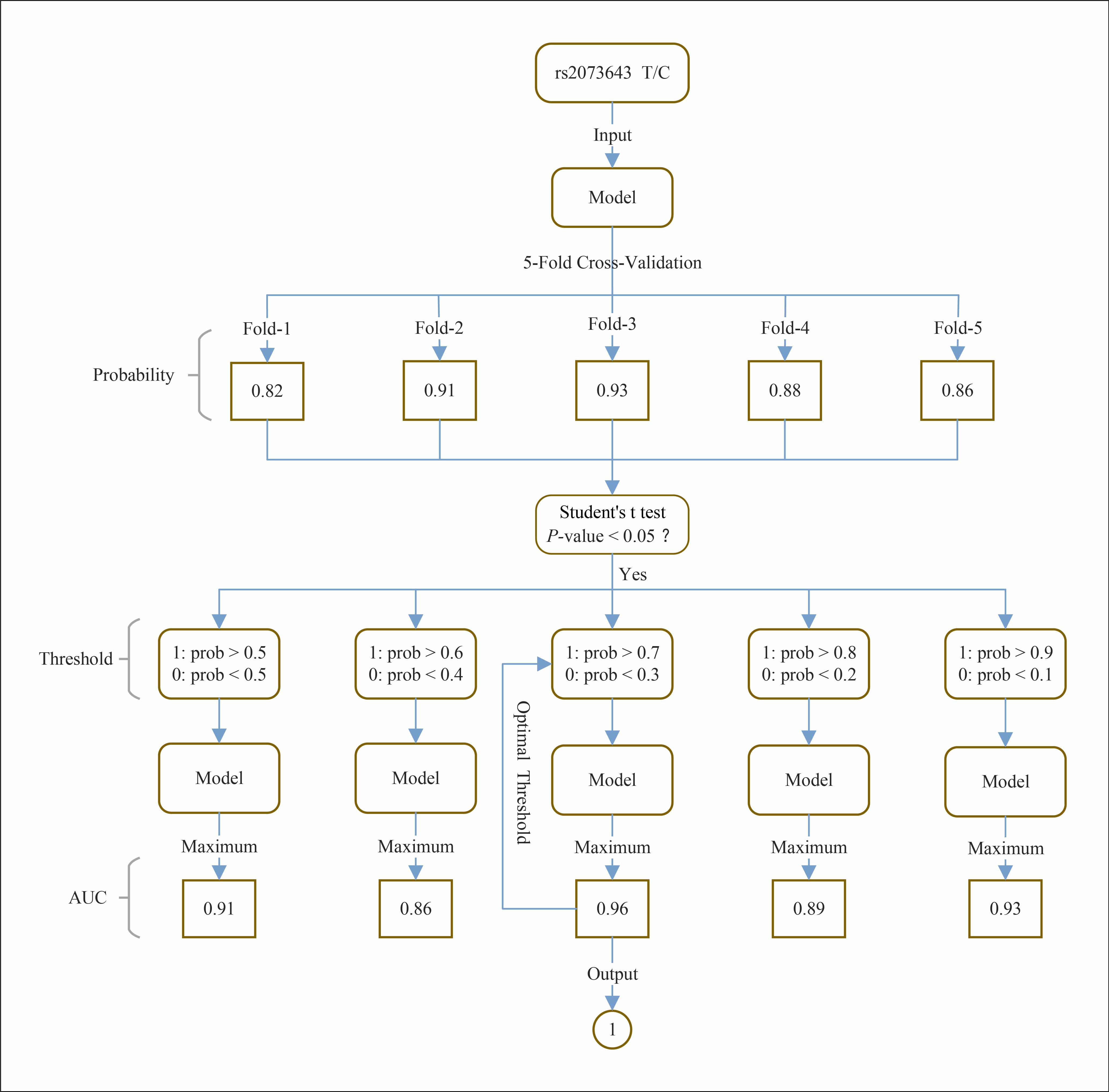


**Supplementary Figure 12.** Workflow for constructing pseudo-label dataset based on GWAS data without ImmuNexUT interactions. For the purpose of illustration, let's input the variant rs2073643 into the model as an example. After performing five-fold cross-validation, the variant will gain five predicted probability values. First, conducting the Student's t test to determine if the differences between these five predicted probability values are statistically significant (*P*-value < 0.05). If the *P*-value of the Student's t-test for the five predicted probability values of this variant is less than 0.05, the variant is retained, otherwise, it is discarded. To find the optimal pseudo-label threshold for this variant, a parameter search is conducted. Using a threshold of 0.5 as a reference, five sets of thresholds (0.5±0, 0.5±0.1, 0.5±0.2, 0.5±0.3, and 0.5±0.4, respectively) are created to pseudo-label this variant. Following the same procedure, apply the above steps to all unlabeled variants. Next, utilizing these variants with pseudo-labeled and the original training dataset to retrain the model and compared the models’ performance to select the best one as sscNOVA. Evaluating the model's performance on the independent testing dataset in terms of AUC. Chose the group of thresholds that yields the highest AUC as the final pseudo-labeling method.


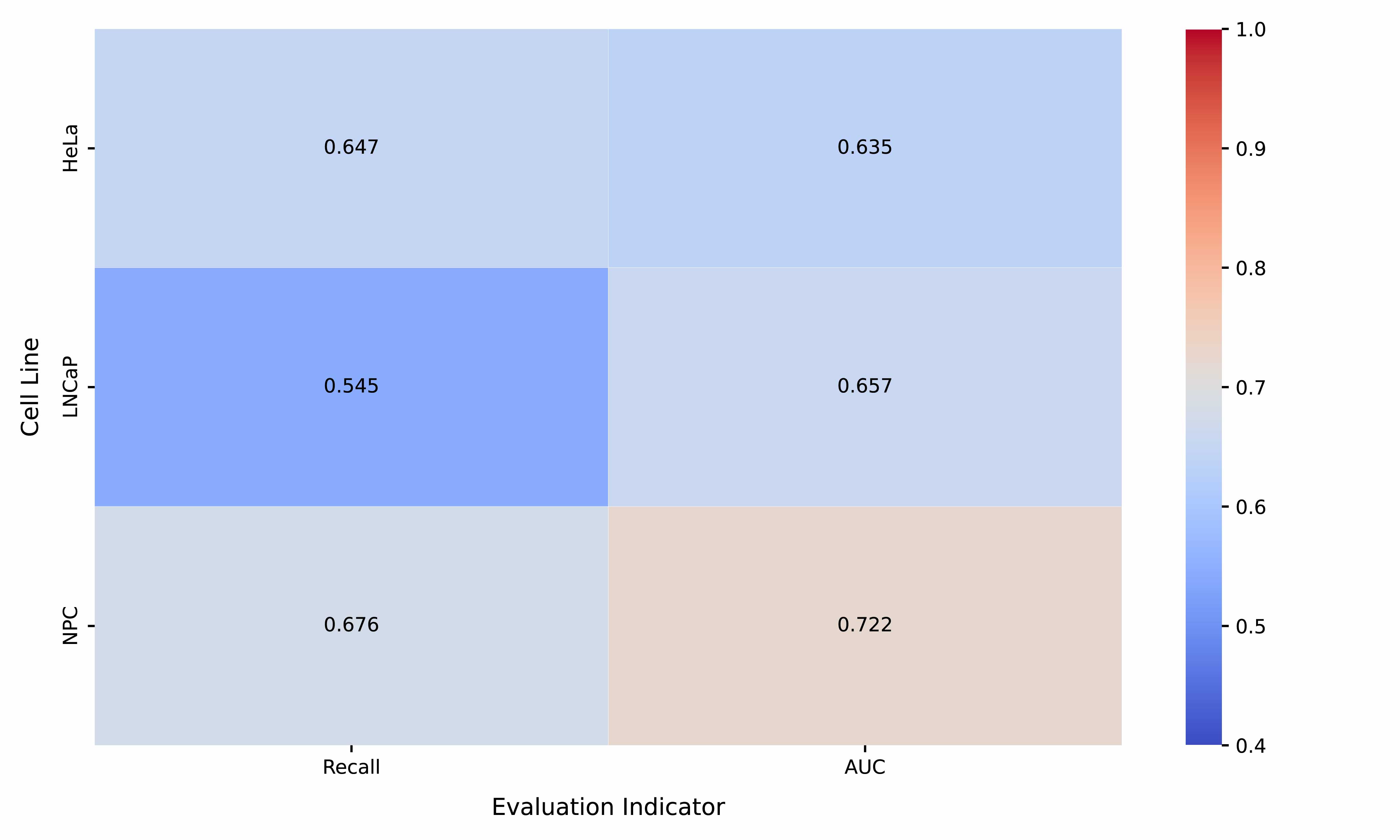


**Supplementary Figure 13.** Heatmap of result for rare or unobserved variants in MPRA assays. Illustrating the predictive performance of the sscNOVA model across HeLa, LNCaP, and NPC cell lines for rare or unobserved variants in MPRA assays. The x axis represents evaluation indicator, the y axis represents the cell lines.


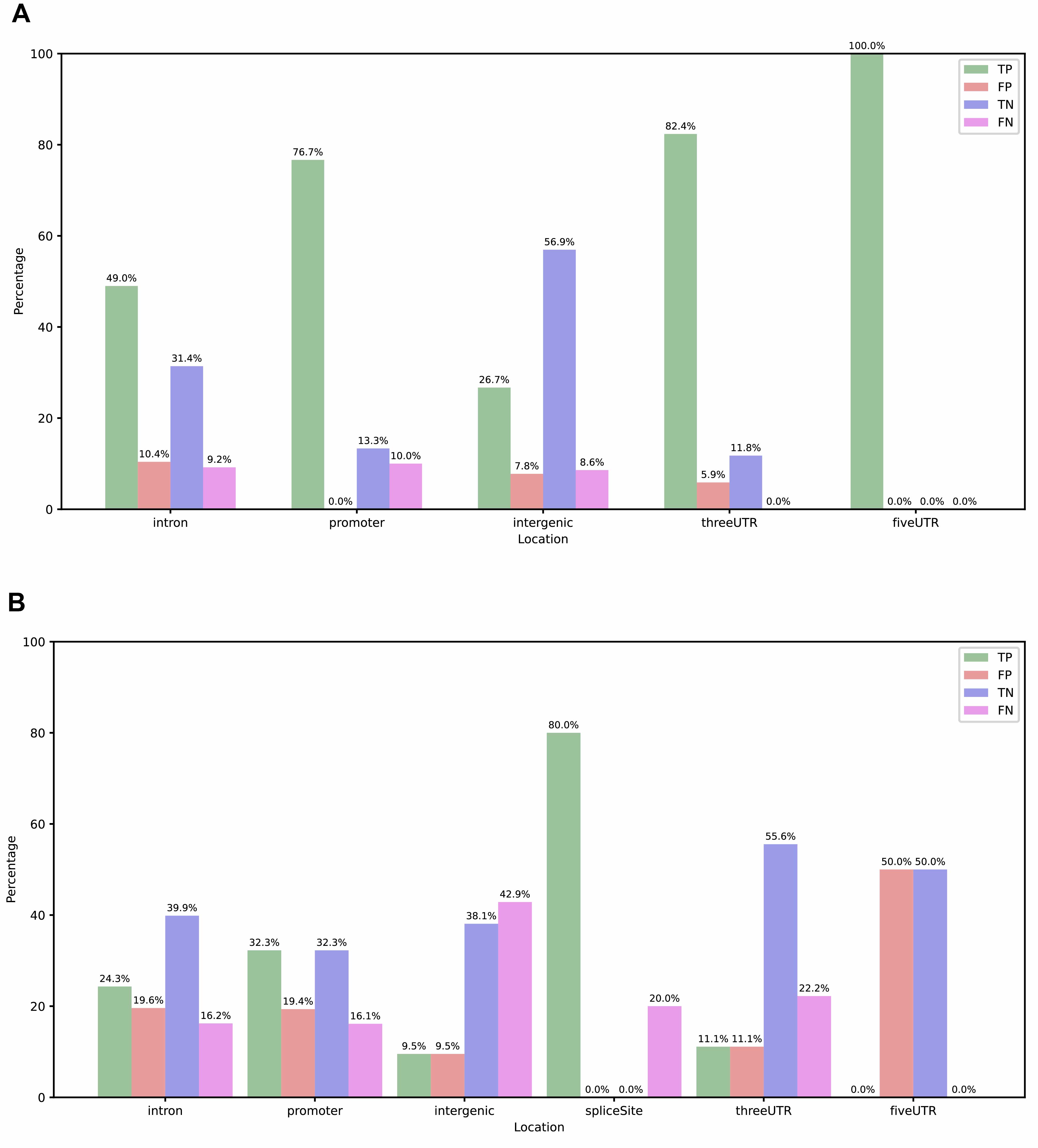


**Supplementary Figure 14.** Variants with successfully identified positional information categorized based on their genomic locations. (A) Variants positional information on the independent testing dataset, these variants are classified into five categories: intron, promoter, intergenic, three prime untranslated region (three UTR), and five prime untranslated region (five UTR). (B) Variants positional information on experimentally curated testing dataset, these variants are classified into six categories: intron, promoter, intergenic, splice site, three prime untranslated region (three UTR), and five prime untranslated region (five UTR). Where the x axis represents categories of genomic location, the y axis represents the percentage of variants. TP represents true positives, FP represents false positives, TN represents true negatives, and FN represents false negatives.
